# Crystal structure of the TLDc domain of human NCOA7-AS

**DOI:** 10.1107/S2053230X21006853

**Published:** 2021-07-28

**Authors:** Mary Arnaud-Arnould, Marine Tauziet, Olivier Moncorgé, Caroline Goujon, Mickaël Blaise

**Affiliations:** a IRIM, CNRS, 34293 Montpellier, France

**Keywords:** viral restriction factors, oxidation resistance, TLDc, human NCOA7-AS

## Abstract

The TLDc [Tre2/Bub2/Cdc16 (TBC), lysin motif (LysM), domain catalytic] domain is associated with oxidation-resistance related functions and is well conserved among eukaryotes. Seven proteins possess a TLDc domain in humans, notably proteins belonging to the oxidation resistance protein (OXR), nuclear receptor coactivator 7 (NCOA7) and TBC1 domain family member 24 (TBC1D24) families. Although the mechanism is unknown, a protective role of TLDc proteins against oxidative stress, notably in the brain, has been demonstrated. Neurobiological disorders caused by mutations in the TLDc domain have also been reported. The human *NCOA7* gene encodes several mRNA isoforms; among these, isoform 4, named *NCOA7-AS*, is up-regulated by type 1 interferon in response to viral infection. NCOA7 and NCOA7-AS both interact with several subunits of the vacuolar proton pump V-ATPase, which leads to increased acidification of the endolysosomal system and consequently impairs infection by viruses that enter their host cells through the endosomal pathway, such as influenza A virus and hepatitis C virus. Similarly to full-length NCOA7, NCOA7-AS possesses a TLDc domain in its C-terminus. Structures of TLDc domains have been reported from zebrafish and fly but not from humans. Here, the expression, purification and crystallization of the TLDc domain from NCOA7 and NCOA7-AS is reported. The crystal structure solved at 1.8 Å resolution is compared with previously solved three-dimensional structures of TLDc domains.

## Abstract 2

The crystal structure of the TLDc domain of the human NCOA7-AS protein, which acts as an interferon-induced antiviral inhibitor, is reported.

## 1. Introduction   

Nuclear receptor coactivator 7 (NCOA7) belongs to the TLDc [Tre2/Bub2/Cdc16 (TBC), lysin motif (LysM), domain catalytic] domain-containing family of proteins. In human and mouse, seven TLDc domain-containing proteins have been reported, among which are oxidation resistance (OXR) proteins 1 and 2 and NCOA7 short and long [alternative start (AS) and full-length (FL), respectively] isoforms (Volkert *et al.*, 2000 ▸; Durand *et al.*, 2007 ▸; Finelli & Oliver, 2017 ▸). NCOA7-FL associates with the estrogen receptor and has been reported to translocate to the nucleus upon estradiol treatment, where it was suggested to act as a transcriptional coregulator (Shao *et al.*, 2002 ▸). In addition, TLDc domain-containing proteins have been shown to play a protective role against oxidative stress, notably in the brain, through an unknown mechanism (Finelli *et al.*, 2016 ▸; Finelli & Oliver, 2017 ▸). The short isoform of NCOA7, NCOA7-AS, does not seem to share this property and is uniquely regulated by type 1 interferon (IFN) via an internal promoter (Yu *et al.*, 2015 ▸).

Additional functions have recently been attributed to NCOA7-AS, which plays a significant role in the IFN-induced control of influenza A virus (IAV). NCOA7-AS notably impairs IAV replication in pulmonary cells through the regulation of V-ATPase activity (Doyle *et al.*, 2018 ▸). As previously shown for NCOA7-FL (Merkulova *et al.*, 2015 ▸), NCOA7-AS interacts with several subunits of the vacuolar V-ATPase, the proton pump responsible for endosomal acidification (Doyle *et al.*, 2018 ▸). NCOA7-AS and NCOA7-FL mostly share the TLDc domain, pinpointing a probable role for this domain in interaction with the V-ATPase. Through an as yet unelucidated mechanism, this interaction leads to greater acidification of the endolysosomal system, which increases antigen degradation and is detrimental to IAV, possibly by irreversibly affecting the ability of haemagglutinin to allow fusion with the host cells (Doyle *et al.*, 2018 ▸). In line with this finding, the *NCOA7* locus has been shown to be important for regulation of V-ATPase function. Interestingly, NCOA7-FL has been shown to interact with the vacuolar V-ATPase in the brain, which enables the correct assembly and transport activity of the proton pump (Castroflorio *et al.*, 2021 ▸). Similar to the NCOA7 isoforms, the OXR1 TLDc protein has also been identified as a V-ATPase partner, suggesting that regulation of the V-ATPase could be a common feature of TLDc family members (Merkulova *et al.*, 2015 ▸). Despite these findings, the function of the TLDc domain is not yet clearly established and it is not known whether this domain is solely responsible for direct interaction with the V-ATPase.

Mutations in genes encoding TLDc domain-containing proteins have been reported in several human diseases (Finelli & Oliver, 2017 ▸). Missense mutations in the TLDc domain found at the C-terminus of the TBC1D24 protein trigger multiple phenotypes, but seem to be particularly linked to epilepsy (Falace *et al.*, 2010 ▸; Lüthy *et al.*, 2019 ▸). Via its TBC domain in the N-terminus, TBC1D24 regulates synaptic vesicle trafficking, which is exerted through the capacity of this domain to interact with small Rab GTPases (Frasa *et al.*, 2012 ▸). However, the function of the TLDc domain of TBC1D24 remains elusive. Little structural information has been gathered on proteins harboring a TLDc domain. The crystal structure of the Skywalker/TBC domain of TBC1D24 has been solved (Fischer *et al.*, 2016 ▸) and two studies have reported crystal structures of the OXR2 and TBC1D24 TLDc domains from *Danio rerio* (Blaise *et al.*, 2012 ▸) and *Drosophila melanogaster* (Lüthy *et al.*, 2019 ▸). The determination of the three-dimensional structure of the TLDc domain from a fly orthologue of human TBC1D24 enabled the pathological mutations linked to human epilepsy to be mapped, and it was proposed that some of these mutations could impair the stability of the protein (Lüthy *et al.*, 2019 ▸), illustrating that structural data on the TLDc domain could be of help in deciphering the impact of genetic mutations. However, to date, no structural information is available for any human TLDc domain.

In this context, and because of the important roles and functions of TLDc domain-containing proteins in various cellular processes and because pathological mutations are found in this domain in human diseases, we engaged in structural studies of the human TLDc domain. Here, we report the crystal structure of the TLDc domain of the human NCOA7-AS protein at high resolution.

## 2. Materials and methods   

### 2.1. Gene cloning   

The TLDc coding sequence of *NCOA7* (encoding amino acids 54–219) was amplified from pRRL.sin.cPPT.SFFV/IRES-puro.WPRE.*NCOA7* variant 6 (Doyle *et al.*, 2018 ▸) using the primers 5′-CGGGGTACCGAGAATCTGTACTTCCAGGGAATGCGGCCCCACAGCGCGC-3′ and 5′-AATTAATTTACTCGAGTCAATCAAATGCCCACACCTCCAG-3′. The PCR fragment was digested and cloned into KpnI/XhoI-digested pET-30 Ek/LIC expression vector (Novagen). The insert is in frame with an S-tag and a His-tag, and a sequence encoding the Tobacco etch virus (TEV) protease cleavage site was inserted upstream of the TLDc coding region to enable tag removal during the purification process.

### 2.2. Protein expression and purification   

The recombinant plasmid pET-30 Ek/LIC::*TLDc* was transformed into the *Escherichia coli* BL21 (DE3) strain resistant to phage T1 (New England Biolabs, Evry, France) carrying pRARE2. One colony was used to inoculate an overnight culture of 500 ml LB medium supplemented with kanamycin (50 µg ml^−1^) and chloramphenicol (34 µg ml^−1^). This culture was diluted in 10 l LB medium supplemented with the two antibiotics. The cells were grown at 289 K to an optical density at 600 nm of 0.8, and protein expression was then induced with 1 m*M* isopropyl β-d-thiogalactopyranoside (IPTG). The culture was grown overnight at 289 K. The cells were harvested by centrifugation at 8200*g* for 20 min and were resuspended in 100 ml buffer *A* (50 m*M* Tris–HCl pH 8, 400 m*M* NaCl, 5 m*M* β-mercaptoethanol, 40 m*M* imidazole, 1 m*M* benzamidine). The cells were disrupted by sonication and cell debris was removed by centrifugation at 28 000*g* for 60 min. The supernatant was loaded at 277 K onto Ni–NTA agarose beads previously equilibrated with buffer *A*. The beads were washed twice with buffer *B* (50 m*M* Tris–HCl pH 8, 1 *M* NaCl, 5 m*M* β-mercaptoethanol, 40 m*M* imidazole, 1 m*M* benzamidine) and elution was performed with buffer *E* (50 m*M* Tris–HCl pH 8, 400 m*M* NaCl, 5 m*M* β-mercapto­ethanol, 500 m*M* imidazole). The eluted protein was incubated with His-tagged TEV protease purified in our laboratory in a 1:100(*w:w*) ratio; the cleavage reaction was performed during dialysis (dialysis-bag cutoff 12–15 kDa) against 1 l dialysis buffer *D* (50 m*M* Tris–HCl pH 8, 100 m*M* NaCl, 5 m*M* β-mercaptoethanol) overnight at 277 K. After dialysis, the proteins were centrifuged for 20 min at 28 000*g* and the supernatant was again loaded at 277 K onto Ni–NTA agarose beads equilibrated with buffer *D*. The TLDc domain without the tag was collected in the flowthrough, concentrated to 5 mg ml^−1^ using a Vivaspin column (10 kDa cutoff), loaded onto a size-exclusion chromatography column (Superdex 75 10/300 GL, GE Healthcare) and eluted with buffer *D*. Following this protocol, 1 mg highly pure protein as judged by a Coomassie Blue-stained denaturing gel was obtained from 1 l culture. Macromolecule-production information is summarized in Table 1 ▸.

### 2.3. Crystallization   

Initial crystallization screening was performed at two protein concentrations: 4.2 and 10.9 mg ml^−1^. The vapor-diffusion method was performed in sitting drops by mixing 0.6 µl protein solution with 0.6 µl reservoir solution using 96-well Swissci MRC plates (Molecular Dimensions, Suffolk, UK) at 291 K. The commercial Structure 1 + 2 (Molecular Dimensions), Index, SaltRx and PEGRx screens (Hampton Research) were assessed. Several hits were obtained and crystal optimization was performed in Swissci 48-Well MRC Maxi Optimization Plates. The best diffraction crystals derived from these optimizations were obtained by mixing 1.5 µl protein solution at 10.9 mg ml^−1^ with 1.5 µl reservoir solution consisting of 0.1 *M* sodium acetate pH 4.5, 32% PEG 300 (Table 2 ▸). The crystals were cryocooled in liquid nitrogen without any cryoprotection prior to data collection.

### 2.4. Data collection and processing   

X-ray data were collected on the ID30A-1/MASSIF-1 beamline at the European Synchrotron Radiation Facility (ESRF), Grenoble, France. The data set was recorded on a PILATUS3 2M detector (Dectris) at a wavelength of 0.965 Å (12.842 keV) and a crystal-to-detector distance of 190.5 mm. A total of 934 images were collected with an exposure time of 0.097 s, a rotation range of 0.15° and full beam transmission. Data were processed, scaled and merged with *XDS* (Kabsch, 2010 ▸) and the data-collection statistics are given in Table 3 ▸.

### 2.5. Structure solution and refinement   

The structure was solved by molecular replacement performed with *Phaser* (McCoy *et al.*, 2007 ▸) from the *Phenix* package (Liebschner *et al.*, 2019 ▸) using the TLDc structure from zebrafish (PDB entry 4acj; Blaise *et al.*, 2012 ▸) as a search model. *Coot* (Emsley *et al.*, 2010 ▸) was used for manual rebuilding, while structure refinement and validation were performed with the *Phenix* package. Because of the high resolution of the data, NCS restraints were not applied during refinement. The statistics for structure refinement are displayed in Table 4 ▸. Figures were prepared with *PyMOL* (http://www.pymol.org).

## 3. Results and discussion   

**Figure 1:**
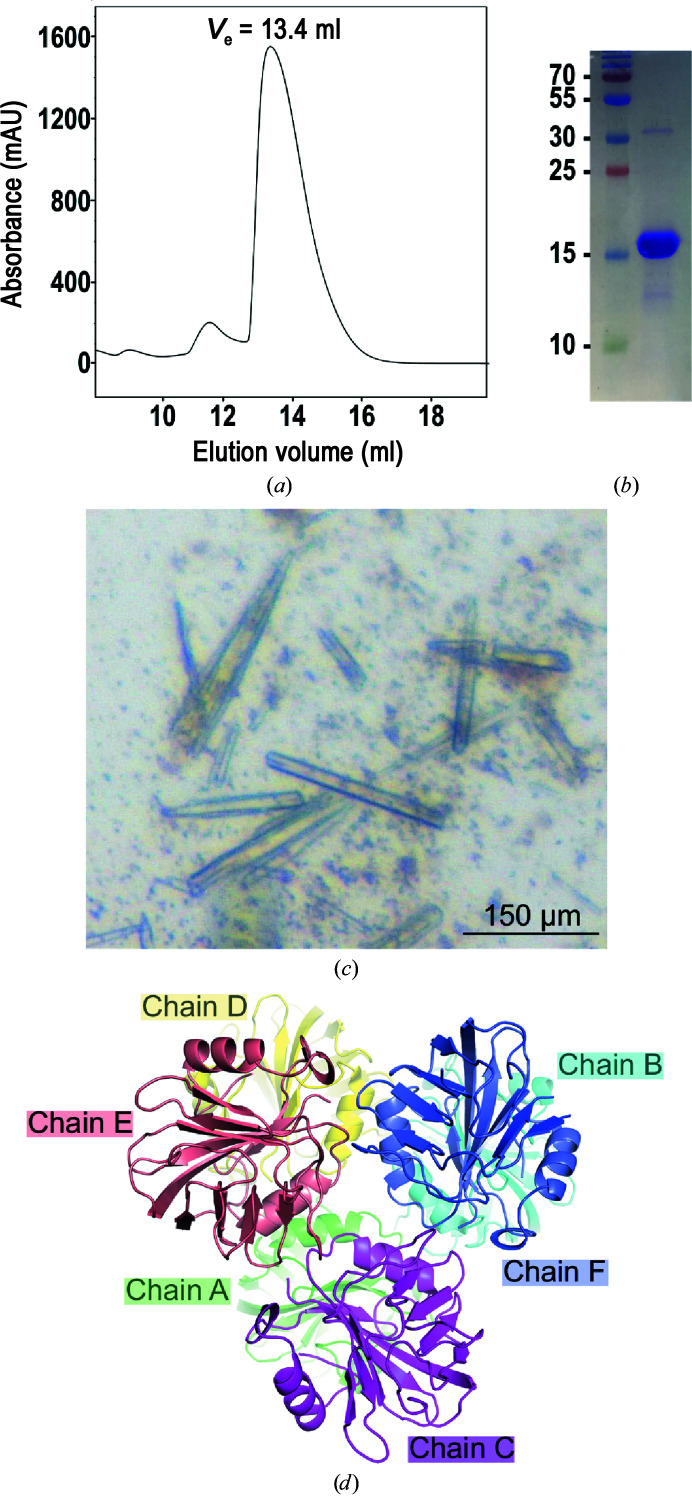
Purification and crystallization of the human TLDc domain. (*a*) Elution profile of the purified TLDc domain on a Superdex 75 10/300 GL Increase column. The elution volume of 13.4 ml attests to the presence of a monomer in solution. (*b*) Coomassie Blue-stained SDS polyacrylamide gel electrophoresis of TLDc performed after the last step of purification by size-exclusion chromatography (10 µg protein; right lane). The left lane contains molecular-mass markers (labelled in kDa). (*c*) Crystals of the TLDc domain obtained in a sitting drop using PEG 300 as a precipitant. Crystals reached their final size (about 100–200 µm) within two days. (*d*) Asymmetric unit composition. Six monomers are present in the asymmetric unit, forming two superposed stable trimers as predicted by the *PISA* server (https://www.ebi.ac.uk/pdbe/pisa/). Chains *A*, *B* and *D* and chains *C*, *E* and *F* form the two assemblies.

As NCOA7-AS plays an important function, notably as an interferon-induced antiviral inhibitor, we aimed to solve its crystal structure. The 219-amino-acid-long NCOA7-AS can be divided into two parts: an N-terminal domain (residues 1–53) followed by the TLDc domain (residues 54–219). We have performed bioinformatic analyses that predicted the first 53 amino acids to be mainly unfolded, with only the presence of two β-strands formed by residues 10–15 and 24–28. Despite numerous efforts, we have so far been unable to purify full-length NCOA7-AS expressed in *E. coli* to homogeneity. NCOA7-AS has a tendency to aggregate and to form large oligomers as judged by size-exclusion chromatography (SEC). We therefore alternatively expressed and purified a truncated form of NCOA7-AS corresponding to the TLDc domain, hereafter referred to as TLDc_Hs_, using *E. coli* as an expression host (Table 2 ▸). TLDc_Hs_ could be purified using a three-step chromatography procedure as a very pure and homogeneous material as attested by SEC (Figs. 1 ▸ *a* and 1 ▸ *b*).

We could crystallize the domain under several conditions using commercial screens. Crystallization-condition optimization led to rod-shaped crystals with a length of about 100–150 µm (Fig. 1 ▸ *c*), which were obtained in 0.1 *M* sodium acetate pH 4.5, 32% PEG 300 (Table 2 ▸). A full X-ray data set could be collected and processed to a resolution of 1.8 Å. The crystals belonged to the orthorhombic space group *P*2_1_2_1_2_1_, with unit- cell parameters as indicated in Table 3 ▸. The Matthews coefficient (*V*_M_) of 2.2 Å^3^ Da^−1^ assumes the presence of 44.5% solvent and six molecules of TLDc in the asymmetric unit (Fig. 1 ▸ *d*).

The TLDc_Hs_ structure was solved by molecular replacement using the TLDc domain (PDB entry 4acj) from *D. rerio* (TLDc_Dr_) as a search model, which shares 62% sequence identity with its human homologue. Six molecules of TLDc_Hs_ were found in the asymmetric unit, as expected from the *V*_M_. The structure was manually rebuilt and refined to *R*_work_ and *R*_free_ values of 0.176 and 0.221, respectively, with rather good geometry, as indicated in Table 4 ▸. Most residues could be rebuilt for the six monomers, except for the first two amino acids of chain *A* and the first three residues at the N-terminus for chains *B*, *C*, *D*, *F* and *E*. Gly175 was not modeled in chain *A* as well as the glycine stretch ranging from 174 to 176 in chain *F*. Residues 146–148 were also disordered in chain *F* but were well ordered in the other chains. Analysis of the crystal packing with the *PISA* server (Krissinel & Henrick, 2007 ▸) revealed the potential existence of a stable complex made of three TLDc_Hs_ monomers within the asymmetric unit (Fig. 1 ▸ *d*). Nonetheless, our SEC analysis demonstrated that the protein behaves only as a monomer in solution (Fig. 1 ▸ *a*), as also described for the TLDc domain from zebrafish (Blaise *et al.*, 2012 ▸).

**Figure 2:**
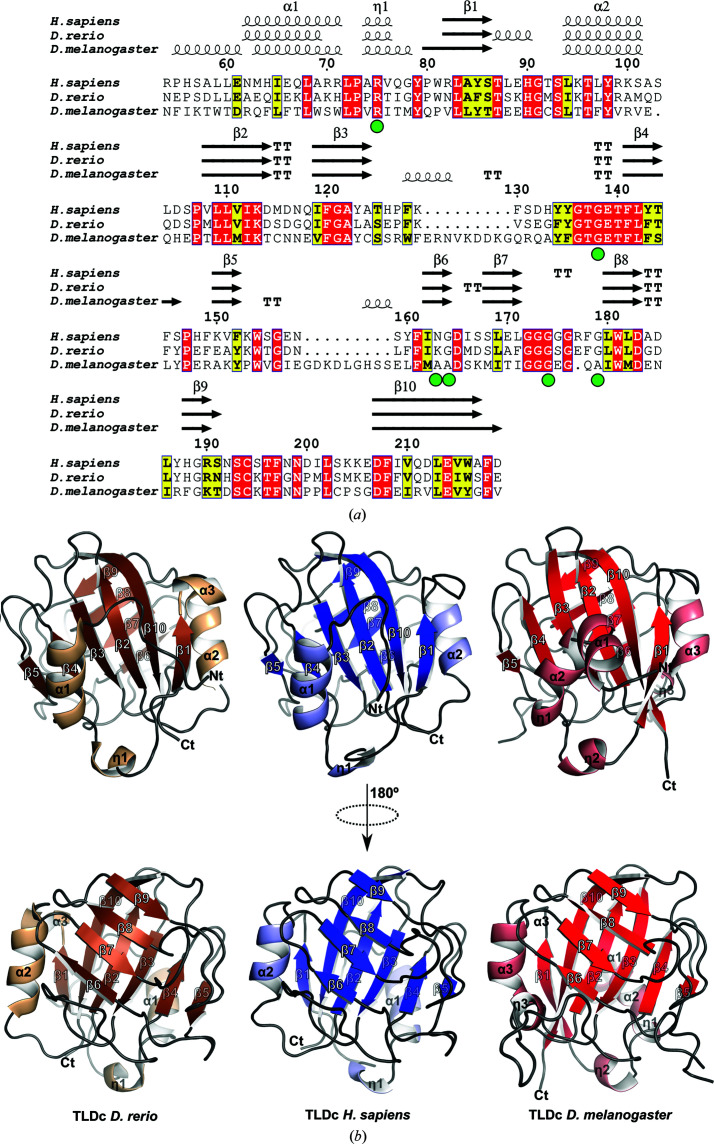
Protein sequences and structural comparisons of the TLDc domains. (*a*) The multiple sequence alignment was performed with *ENDscript* (Robert & Gouet, 2014 ▸) and adjusted manually. The secondary structures (α, α-helix; β, β-strand, η, 3_10_-helix; TT, turn) of the three TLDc structures extracted from the crystal structures are indicated above the alignment. The green spheres below the alignment indicate the position of missense mutations that are found in the TLDc domain of TBC1D24 and are associated with human diseases. (*b*) Structural comparison of the three TLDc domains from *D. rerio* (PDB entry 4acj; left; brown), *H. sapiens* (PDB entry 7obp; middle; blue) and *D. melanogaster* (PDB entry 6r82; right; red).

The overall structure of TLDc_Hs_ is globular and consists of two α-helices in the N-terminus and ten β-strands forming two antiparallel β-sheets. The two sheets, organized as a central pseudo-orthogonal β-sandwich, are made by strands β1, β2, β3, β4, β5 and β10 and strands β6, β7, β8 and β9, respectively. The N-terminal part of β10 interacts with β9 and to this extent contributes to forming the second β-sandwich. The fold is similar to those of the two previously solved TLDc structures from *D. rerio* OXR2 (TLDc_Dr_) and *D. melanogaster* TBC1D24 (TLDc_Dm_). TLDc_Hs_ shares 62% sequence identity with TLDc_Dr_ (Fig. 2 ▸ *a*) and the two structures display an r.m.s.d. of 0.7 Å when superposed over 164 C^α^ atoms. Few structural differences could be observed. TLDc_Hs_ does not possess an α-helix after strand β1 as seen in TLDc_Dr_ (Fig. 2 ▸ *b*). TLDc_Hs_ and TLDc_Dm_ are more distant as they present only 38% sequence identity (Fig. 2 ▸ *a*), and superimposition of the two structures leads to an r.m.s.d. of 1.1 Å over 144 residues. The N-terminal sequences are not well conserved in the three proteins compared with the rest of the sequence, which is reflected by a few differences at the three-dimensional level. TLDc_Dm_ possesses one extra helix (α2) in the N-terminus that is not seen in TLDc_Hs_ or TLDc_Dr_. Finally, noticeable differences are found as two extended loops between β3 and β4 and β5 and β6 in TLDc_Dm_.

Several missense mutations have been reported in the TLDc domain from TBC1D24 (Table 5 ▸) that are linked to human diseases (Falace *et al.*, 2010 ▸; Balestrini *et al.*, 2016 ▸; Wang *et al.*, 2019 ▸; Lüthy *et al.*, 2019 ▸; Muona *et al.*, 2015 ▸; Uzunhan & Uyanik, 2020 ▸; Atli *et al.*, 2018 ▸). The three-dimensional structure of TLDc_Hs_ therefore offers the possibility to map these mutations onto the domain. We performed mapping of these mutations onto TLDc_Hs_ (Figs. 2 ▸ *a* and 3 ▸) and assessed the conservation of the residues with missense mutations mapped onto TLDc_Dm_ from fly TBC1D24 as described previously by Lüthy *et al.* (2019 ▸). The pathological Arg360His or Arg360Leu mutation found in the human TBC1D24 protein is situated in 3_10_-helix η1; the equivalent residue in TLDc_Hs_ from NCOA7-AS is Arg75. Arg75 is involved in a salt-bridge interaction with the side chain of the highly conserved Glu139 (not shown), and mutation(s) breaking this interaction might therefore destabilize the long loop spanning between strands β4 and β5 (Figs. 2 ▸ *a* and 3 ▸). The reported Gly428Arg mutation corresponds to Gly138 in human TLDc and is situated just before strand β4. The Ala500 residue mutated to Val in human TBC1D24 is not conserved in TLDc_Hs_ from NCOA7-AS, where Asn163 is instead found. Despite this lack of residue conservation, both amino acids are part of strand β6. Gly501 in TBC1D24 and the equivalent Gly164 in TLDc_Hs_ are also part of strand β6. Mutation(s) in a structured region such as strand β6 will probably affect the folding of this strand. The Gly511 residue mutated to Arg matches Gly174 in TLDc_Hs_ and is situated in a loop formed by a five-glycine stretch between strands β7 and β8. Finally, Ala515, which has been reported to be mutated to Val in several studies, is not conserved in TLDc_Hs_ as Gly179 is instead found. Nonetheless, both residues are at the beginning of strand β8, and these mutations could destabilize the integrity of this strand. The missense mutations found in the TLDc domain of human TBC1D24 which are linked to neurological disorders are all found at structurally conserved positions. It is indeed clear that the Gly511Arg and Gly501Arg mutations as well as the Arg360His/Leu, Ala500Val and Ala515Val mutations could disturb the folding of the TLDc domain and potentially destabilize the protein because of the nature of the amino-acid substitution and/or its position in the structurally conserved regions. This analysis is therefore in strong agreement with the previously proposed effect on protein stability of the TLDc domain induced by pathological mutations found in TBC1D24 (Lüthy *et al.*, 2019 ▸).

To conclude, this structural work strongly attests to the high protein similarity between TLDc domains from vertebrates as well as, albeit slightly more distantly, that from an arthropod. Although this was expected from the high sequence identity that is shared between these proteins, reporting the first crystal structure of a human TLDc domain is notably of interest because of the versatile roles of TLDc proteins and also because pathological mutations affect this domain.

## Supplementary Material

PDB reference: human TLDc domain, 7obp

## Figures and Tables

**Figure 3:**
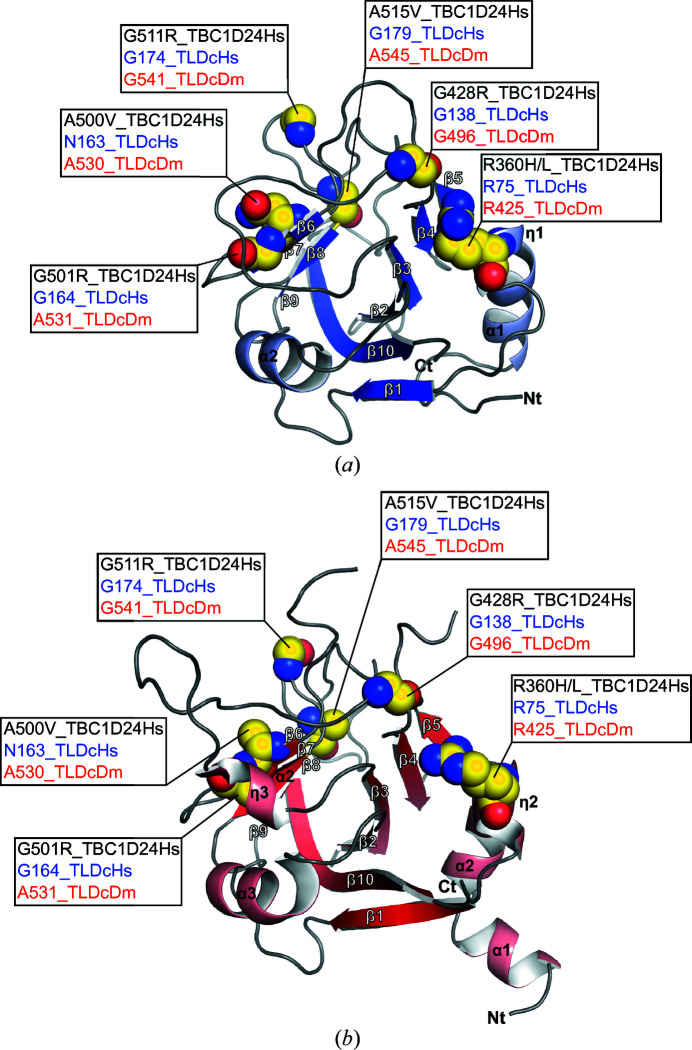
Structural mapping of the pathological mutations found in the human TLDc domain from TBC1D24. The mutations are reported in the crystal structures of TLDc_Hs_ from NCOA7-AS (*a*) and TLDc_Dm_ from fly TBC1D24 (*b*). The human pathological missense mutations reported in TBC1D24 are indicated in black, while the corresponding residues are in blue for the TLDc_Hs_ structure and in red for the TLDc_Dm_ structure. Residues corresponding to the mutations are shown as yellow spheres.

**Table 1:** Macromolecule-production information

Source organism	*H. sapiens*
DNA source	pRRL.sin.cPPT.SFFV/IRES-puro.WPRE.*NCOA7* variant 6†
Forward primer‡	5′-CGG**GGTACC** *GAGAATCTGTACTTCCAGGGA*ATGCGGCCCCACAGCGCGC-3′
Reverse primer§	5′-AATTAATTTA**CTCGAG**TCAATCAAATGCCCACACCTCCAG-3′
Cloning vector	pET-30 Ek/LIC
Expression vector	pET-30 Ek/LIC
Expression host	*E. coli* BL21 (DE3) transformed with the pRARE2 plasmid
Complete amino-acid sequence of the construct produced	MHHHHHHSSGLVPRGSGMKETAAAKFERQHMDSPDLGTENLYFQGMRPHSALLENMHIEQLARRLPARVQGYPWRLAYSTLEHGTSLKTLYRKSASLDSPVLLVIKDMDNQIFGAYATHPFKFSDHYYGTGETFLYTFSPHFKVFKWSGENSYFINGDISSLELGGGGGRFGLWLDADLYHGRSNSCSTFNNDILSKKEDFIVQDLEVWAFD
Complete amino-acid sequence of the construct after TEV cleavage	GMRPHSALLENMHIEQLARRLPARVQGYPWRLAYSTLEHGTSLKTLYRKSASLDSPVLLVIKDMDNQIFGAYATHPFKFSDHYYGTGETFLYTFSPHFKVFKWSGENSYFINGDISSLELGGGGGRFGLWLDADLYHGRSNSCSTFNNDILSKKEDFIVQDLEVWAFD

† Doyle *et al.* (2018 ▸).

‡ Letters in bold indicate the KpnI restriction site and those in italics indicate the sequence encoding the Tobacco etch virus protease cleavage site.

§ Letters in bold indicate the XhoI restriction site.

**Table 2:** Crystallization

Method	Vapor diffusion in sitting drops
Plate type	Swissci 48-Well MRC Maxi Optimization Plates
Temperature (K)	291
Protein concentration (mg ml^−1^)	10.9
Buffer composition of protein solution	50 m*M* Tris–HCl pH 8, 100 m*M* NaCl, 5 m*M* β-mercaptoethanol
Composition of reservoir solution	0.1 *M* sodium acetate pH 4.5, 32% PEG 300
Volume and ratio of drop	1.5 µl protein solution + 1.5 µl reservoir solution
Volume of reservoir (µl)	250

**Table 3:** Data collection and processing Values in parentheses are for the outer shell.

Diffraction source	ID30A-1, ESRF
Wavelength (Å)	0.965
Temperature (K)	100
Detector	PILATUS3 2M
Crystal-to-detector distance (mm)	190.5
Rotation range per image (°)	0.15
Total rotation range (°)	140
Exposure time per image (s)	0.097
Space group	*P*2_1_2_1_2_1_
*a*, *b*, *c* (Å)	68.41, 107.06, 146.44
α, β, γ (°)	90, 90, 90
Mosaicity (°)	0.048
Resolution range (Å)	44.4–1.8 (1.86–1.80)
Total No. of reflections	518696 (47788)
No. of unique reflections	100055 (9863)
Completeness (%)	99.79 (99.78)
Multiplicity	5.2 (4.8)
〈*I*/σ(*I*)〉	10.10 (1.44)
Wilson *B* factor (Å^2^)	19.8
*R*_meas_	0.147 (1.14)
CC_1/2_	0.997 (0.58)

**Table 4:** Structure refinement Values in parentheses are for the outer shell.

Resolution range (Å)	44.4–1.8 (1.86–1.80)
Reflections used in refinement	100033 (9861)
Reflections used for *R*_free_	2000 (197)
*R*_work_	0.176 (0.273)
*R*_free_	0.221 (0.334)
No. of non-H atoms	
Total	9256
Macromolecules	8157
Solvent	1099
Protein residues	984
R.m.s.d., bond lengths (Å)	0.005
R.m.s.d., angles (°)	0.80
Ramachandran favored (%)	97.20
Ramachandran allowed (%)	2.80
Ramachandran outliers (%)	0
Rotamer outliers (%)	0.46
Clashscore	2.44
Average *B* factor (Å^2^)	
Overall	27.88
Macromolecule	26.83
Solvent	35.64
PDB code	7obp

**Table 5:** Comparison of the pathological mutations and associated syndromes found in the TLDc domain from human TBC1D24 with the TLDc domain from *D. melanogaster* TBC1D24 (red) and the TLDc domain from human NCOA7-AS (blue) The residues that differ between the TLDc domains are highlighted in bold.

Pathological mutations in the TLDc domain of human TBC1D24 and associated diseases		Residue in TBC1D24 TLDc_Dm_	Corresponding residue in NCOA7-AS TLDc_Hs_	References
**Arg360His/Leu**	RE-EID (Rolandic epilepsy–writer’s cramp-exercise induced dystonia), progressive myoclonus epilepsy	Arg425	Arg75	Lüthy *et al.* (2019 ▸), Muona *et al.* (2015 ▸)
**Gly428Arg**	RE-EID, DOORS (deafness, onychodystrophy, osteodystrophy and mental retardation syndrome)	Gly496	Gly138	Lüthy *et al.* (2019 ▸), Atli *et al.* (2018 ▸)
**Ala500Val**	RE-EID, focal motor seizures involving the face (with F229S or S473Rfs*43)	Ala530	**Asn163**	Lüthy *et al.* (2019 ▸), Balestrini *et al.* (2016 ▸), Uzunhan & Uyanik (2020 ▸)
**Gly501Arg**	RE-EID	**Ala531**	Gly164	Lüthy *et al.* (2019 ▸)
**Gly511Arg**	RE-EID	Gly541	Gly174	Lüthy *et al.* (2019 ▸)
**Ala515Val**	RE-EID, familial infantile myoclonic epilepsy, impaired neurite growth and length (with Asp147His)	Ala545	**Gly179**	Lüthy *et al.* (2019 ▸), Falace *et al.* (2010 ▸)
